# ROR functions as a ceRNA to regulate Nanog expression by sponging miR-145 and predicts poor prognosis in pancreatic cancer

**DOI:** 10.18632/oncotarget.6450

**Published:** 2015-12-02

**Authors:** Song Gao, Peng Wang, Yongqiang Hua, Hao Xi, Zhiqiang Meng, Te Liu, Zhen Chen, Luming Liu

**Affiliations:** ^1^ Department of Integrative Oncology, Fudan University Shanghai Cancer Center, Shanghai, China; ^2^ Department of Oncology, Shanghai Medical College, Fudan University, Shanghai, China; ^3^ Department of Pathology, Shanghai Tenth People's Hospital, Tongji University School of Medicine, Shanghai, China; ^4^ Shanghai Geriatric Institute of Chinese Medicine, Longhua Hospital, Shanghai University of Traditional Chinese Medicine, Shanghai, China

**Keywords:** incRNA, ROR, microRNA, cancer stem cells, Nanog

## Abstract

lncRNAs have emerged as key regulators of tumor development and progression. ROR is a typical lncRNA that plays important regulatory roles in the pathogenesis and progression of tumors. Nevertheless, current understanding of the involvement of ROR in pancreatic adenocarcinoma tumorigenesis remains limited. In this study, we measured ROR in 61 paired cancerous and noncancerous tissue samples by qRT-PCR and investigated the biological role of ROR on the phenotypes of pancreatic cancer stem cells (PCSCs) *in vitro* and *in vivo*. The effects of ROR on PCSCs were studied by RNA interference approaches *in vitro* and *in vivo*. Insights of the mechanism of competitive endogenous RNAs (ceRNAs) were gained from bioinformatic analysis, luciferase assays and RNA binding protein immunoprecipitation. The positive ROR/Nanog interaction was identified and verified by immunohistochemistry assay. Compared with adjacent non-tumor tissues, ROR was up-regulated in most tumor tissues. Knockdown of ROR by RNA interference in PCSCs inhibited proliferation, induced apoptosis and decreased migration. Moreover, ROR silencing resulted in significantly decreased tumourigenicity of PCSCs in nude mice than controls. In particular, ROR may act as a ceRNA, effectively becoming a sink for miR-145, thereby activating the derepression of core transcription factors Nanog. In conclusions, we demonstrated that decreased ROR expression could inhibit cell proliferation, invasion, and tumourigenicity by modulating Nanog. Therefore, ROR is a potential novel prognostic marker to predict the clinical outcome of pancreatic cancer patients after surgery and may be a rational target for therapy.

## INTRODUCTION

Pancreatic cancer displays one of the highest malignancy rates among tumors, with an extremely high mortality rate [1]. As the early diagnosis of pancreatic cancer is difficult, patients are frequently at an intermediate or advanced stage when diagnosed [2–6]. In addition, the mechanism of pathogenesis in pancreatic cancer remains unclear, and there are currently no effective therapies or drugs.

In recent years, non-coding RNAs have been shown to play a crucial role in regulating cell fate, for example, in embryonic stem cells [7–10]. Non-coding RNAs are characterized in two distinct groups: miRNAs and lncRNAs [8]. LncRNAs are long (> 200 nucleotides) RNA molecules that are widely transcribed in mammalian cell genomes [11]. Although lncRNA and miRNA belong to the same non-coding RNA family, the structures contained within lncRNA sequences, and their processing, are far more complex [11, 12]. Some lncRNAs can bind to specific microRNAs to competitively activate the expression of targeted gene [12–16]. One such lncRNA is HOX Transcript Antisense Intergenic RNA or ROR.

In this study, we observe that ROR was substantially overexpressed in pancreatic cancer tissues and investigate the biological role of ROR on the phenotypes of pancreatic cancer cells *in vitro* and *in vivo*. RoR has been reported to act as an endogenous sponge of miR-145 in hESCs [16]. We assume that it had similar effects in PCSCs. Therefore we propose that miRNA-145 competitively decreases ROR and Nanog expression through a ‘sponge’ effect, and inhibits the proliferation, invasion and tumourigenicity of PCSCs, thus playing an oncogenic role in pancreatic pathogenesis. The present work provides an evidence for a positive ROR/Nanog correlation and the crosstalk between miR-145, ROR and Nanog, shedding new light on the potential therapeutic target in pancreatic cancer.

## RESULTS

### ROR upregulation is associated with poor prognosis in pancreatic cancer

**Figure 1:**
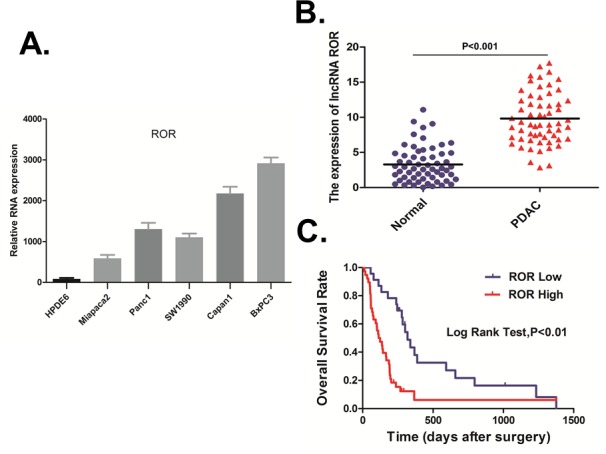
ROR upregulation is associated with poor prognosis in pancreatic cancer **A.** We evaluated the expression of ROR in five pancreatic cancer-derived cell lines (PANC-1, Capan-1, Mia PaCa-2, BxPC-3, and SW1990) and in immortalized human pancreatic ductal epithelial cells (HPDE6) by qRT-PCR. The result indicated that all pancreatic cancer cell lines exhibited higher levels of ROR compared with the non-tumoral pancreatic cell line, HPDE6, with the highest expression observed in BxPC-3 cells. **B.** We then analyzed ROR expression in 61 paired resected samples by qRT-PCR. Compared with adjacent non-tumor tissues, ROR was up-regulated in most PDAC tissues. **C.** We then analyzed ROR expression for associations with clinicopathological parameters, such as gender, age, Tumor Location, Tumor Size, Nodal Metastasis, CA19-9, TNM Stage, Tumor differentiation. The data indicated that ROR expression was positive associated with Tumor Size (*p* < 0.05). High ROR expression was also associated with poor overall survival.

We evaluated the expression of ROR in five pancreatic cancer-derived cell lines (PANC-1, Capan-1, MiaPaCa-2, BxPC-3, and SW1990) and in immortalized human pancreatic ductal epithelial cells (HPDE6) by qRT-PCR. The result indicated that all pancreatic cancer cell lines exhibited higher levels of ROR compared with the non-tumoral pancreatic cell line, HPDE6, with the highest expression observed in BxPC-3 cells (Figure 1A). We then analyzed ROR expression in 61 paired resected samples by qRT-PCR. Compared with adjacent non-tumor tissues, ROR was up-regulated in most pancreatic duct adenocarcinoma (PDAC) tissues (Figure 1B). We then analyzed ROR expression for associations with clinicopathological parameters, such as gender, age, tumor location, tumor size, nodal metastasis, CA19-9, TNM Stage, tumor differentiation (Supplemental Table 1). The data indicated that ROR expression was positive associated with Tumor Size (*p* < 0.05). High ROR expression was also associated with poor overall survival (*p* < 0.01, Figure 1C).

### ROR and miR-145 expression are negatively correlated

**Figure 2:**
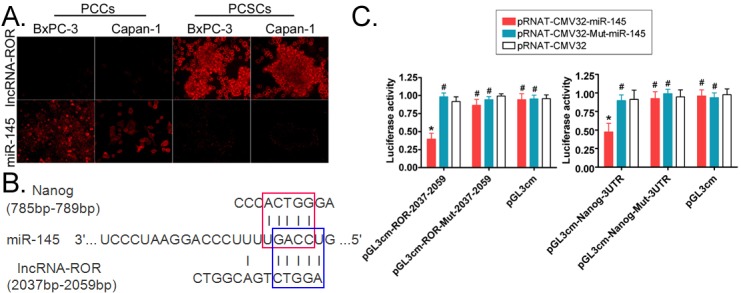
ROR and Nanog share a common miR-145 binding site **A.** fluorescence *in situ* hybridization experiments show that ROR and miR-145 display opposite expression levels in pancreatic cancer cells (PCCs) compared to pancreatic cancer stem cells (PCSCs). Original magnification, 200×. **B.** Bioinformatics analysis revealed that ROR and Nanog share a common miR-145 binding site. The red box represents the binding site for miR-145 on the Nanog mRNA 3′UTR, and the blue box represents the binding site for miR-145 on ROR. **C.** The luciferase reporter assay results showed that when simultaneously overexpressing miR-145 and the ROR 2055 bp-2059 bp sequence in the same cell line, the luciferase activity was significantly lower than that of the control group. Similarly, when simultaneously overexpressing miR-145 and the Nanog mRNA 3′UTR in the same cell line, the Luciferase activity was significantly lower than that of the control group (pRNT-CMV32) (* *p* < 0.05; # *p* > 0.05; *n* = 3).

Fluorescence *in situ* hybridization (FISH) was performed and revealed that the expression of ROR in PCSCs was significantly higher than in pancreatic cancer cells (PCCs), whereas miR-145 expression was higher in PCCs than PCSCs (Figure 2A). We also analyzed the ROR and miR-145 in serial sections of tissues by FISH. The results were in agreement with the expression in PCSCs and PCCs (Supplemental Figure 1), ROR silencing resulted in increased expression of miR-145. So we further confirm that the expression of ROR and miR-145 are negatively correlated.

### MiR-145 competitively binds to ROR and Nanog

A bioinformatics analysis showed that there are conserved binding sites for miR-145 on both ROR and the 3′UTR of Nanog mRNA. MiR-145 can complementarily bind to the ROR sequence between 2055 bp and 2059 bp, and its sequence is also complementary to the 3′UTR sequence of Nanog mRNA between 785 bp and 789 bp (Figure 2B). Using a luciferase reporter assay, we found that the luciferase activity was significantly lower than the control group (*p* < 0.05) when miR-145 and the ROR 2055 bp-2059 bp sequence were simultaneously overexpressed in the same cell line (Figure 2C). Similarly, the luciferase activity was significantly lower than the control group (*p* < 0.05) when miR-145 and the Nanog mRNA 3′UTR were simultaneously overexpressed in the same cell line (Figure 2C). These results suggest that miR-145 can induce post-transcriptional silencing of its targeted genes by binding to the Nanog mRNA 3′UTR or ROR specific sites. Further, the results also suggest that there is competition for miR-145 between ROR and Nanog.

### SiRNA interference of ROR expression results in the inhibition of *in vitro* proliferation and invasion of PCSCs

We chose two siRNAs, siROR-1 and siROR-2, for the construction of the pGIPZROR-shRNA plasmids so as to exclude off-target effects. The pGIPZ ROR-shRNA vectors with an EGFP marker were then packaged into lentiviruses and transduced into human BxPC3 and Capan1 pancreatic cancer stem cells. Then we detected ROR expression by qRT-PCR. The results indicated that the transfection efficiency of siROR-1 and siROR-2 is higher (Supplemental Figure 2).

**Figure 3:**
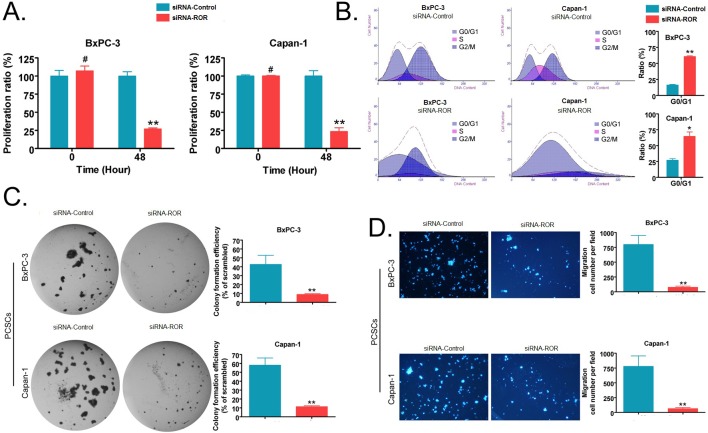
Silencing endogenous ROR expression by siRNA can effectively inhibit the *in vitro* proliferation, invasion and cell cycle progression of pancreatic cancer stem cells **A.** The CCK-8 experiment showed that the *in vitro* proliferation rate of siRNA-ROR-transfected PCSCs was significantly lower than that of PCSCs transfected with a random sequence (siRNA-Control) (** *p* < 0.01; # *p* > 0.05; *n* = 3). **B.** Cell cycle results detected by flow cytometry showed that the proportion of G0/G1 phase of siRNA-ROR-transfected PCSCs was significantly higher than that of PCSCs transfected with siRNA-Control (** p < 0.01; * *p* < 0.05; *n* = 3). **C.** The soft agar colony formation assay results showed that the colony formation rate of siRNA-ROR-transfected PCSCs in soft agar was significantly lower than that of siRNA-Control-transfected PCSCs (** *p* < 0.01; *n* = 3). **D.** The transwell migration invasion assay results showed that the number of cells that invaded the membrane in the siRNA-ROR-transfected PCSC group was significantly less than that of the siRNA-Control-transfected PCSCs (** *p* < 0.01; *n* = 3).

Using a cell proliferation assay, we observed that the i*n vitro* proliferation rate of PCSCs (from the BxPC-3 and Capan-1 cell lines) transfected with siRNA targeting ROR was significantly lower than PCSCs transfected with a scrambled siRNA sequence (siRNA-Control) (*p* < 0.01, Figure 3A). Next, we analyzed the proportion of cells in various stages of the cell cycle using flow cytometry, the results indicate that silencing of ROR results in the blockage of PCSCs at the G0/G1 phase in the cell cycle and inhibits their proliferation (*p* < 0.01, Figure 3B). Further, the soft agar colony formation assay results showed that the colony formation rate of PCSCs transfected with siRNA-ROR in soft agar was significantly lower than in siRNA- Control group (*p* < 0.01, Figure 3C). At the same time, the transwell migration invasion assay results showed that the number of cells that invaded under the membrane in PCSCs transfected with siRNA-ROR was significantly less than those transfected with siRNA- Control (*p* < 0.01, Figure 3D). These experiments show that silencing the expression of ROR can effectively inhibit the *in vitro* proliferation and invasive ability of PCSCs.

### Inhibition of ROR expression by siRNA can promote upregulated expression of endogenous miR-145 and silencing of nanog expression

**Figure 4:**
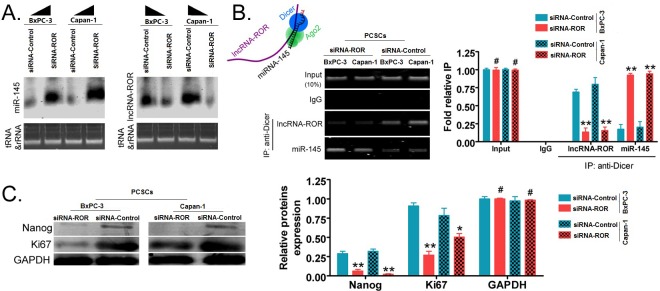
Inhibition of ROR expression by siRNA can promote endogenous miR-145 silencing of Nanog expression in PCSCs **A.** the Northern blot experiment showed that the ROR hybridization signal in the siRNA-ROR transfection group was significantly weaker than in the siRNA-Control-transfected PCSCs. However, the miR-145 hybridization signal in the siRNA-ROR transfection group was significantly stronger than in the siRNA-Control transfection group. **B.** the RNA immunoprecipitation (RIP)-PCR experiment for detecting mRNA binding to the Dicer enzyme. In siRNA-ROR-transfected PCSCs, the cross-linking signal of miR-145 to the Dicer enzyme was stronger than in the siRNA-Control-transfected PCSCs. The ROR signal was barely detected in siRNA-ROR-transfected PCSCs. (** *p* < 0.01; # *p* > 0.05; *n* = 3). **C.** western blot experiments showed that in siRNA-ROR-transfected PCSCs, the expression level of Nanog and Ki67 proteins was significantly lower than in the siRNA-Control transfection group (** *p* < 0.01; * *p* < 0.05; # *p* > 0.05; *n* = 3).

Northern blot was used to detect RNA expression levels of ROR and miR-145. The ROR hybridization signal was weaker in the siRNA-ROR-transfected PCSCs than in the siRNA- Control -transfected cells. However, the miR-145 hybridization signal in the siRNA-ROR-transfected cells was significantly stronger than in the siRNA-Control-transfected PCSCs (Figure 4A). Next, RNA immunoprecipitation (RIP)-PCR experiments were used to detect the protein-nucleic acid complex of RNA bound to the Dicer enzyme, i.e. the enzyme responsible for miRNA cleavage and thus, suppression of gene expression. When PCSCs overexpress siRNA-ROR, the cross-linking signal of miR-145 to the Dicer enzyme was strong, while the nucleic acid signal of ROR was barely detected (Figure 4B). However, in siRNA-Control-PCSCs, there was a PCR band corresponding to ROR in the Dicer enzyme-nucleic acid complex, whereas the miR-145 PCR signal was very weak (Figure 4B). The results were in agreement with the expression in PCSCs and PCCs, ROR silencing resulted in increased expression of miR-145. So we further confirm that the expression of ROR and miR-145 are negatively correlated in pancreatic cancer tissue samples(Supplemental Figure 1).

Western blotting revealed that in siRNA-ROR-transfected PCSCs, the protein expression level of the embryonic stem cell pluripotent transcription factor Nanog was significantly lower than in the siRNA- Control-transfected group (*p* < 0.01, Figure 4C). The protein expression level of the cell proliferation factor Ki67 was also significantly lower in siRNA-ROR-transfected PCSCs than in the siRNA- Control-transfected group (*p* < 0.05, Figure 4C). Together these results demonstrate that down-regulation of ROR expression can increase the expression of endogenous miR-145 and silence of Nanog expression.

### Inhibition of endogenous ROR expression by siRNA can reduce the *in vivo* tumourigenicity of PCSCs

**Figure 5:**
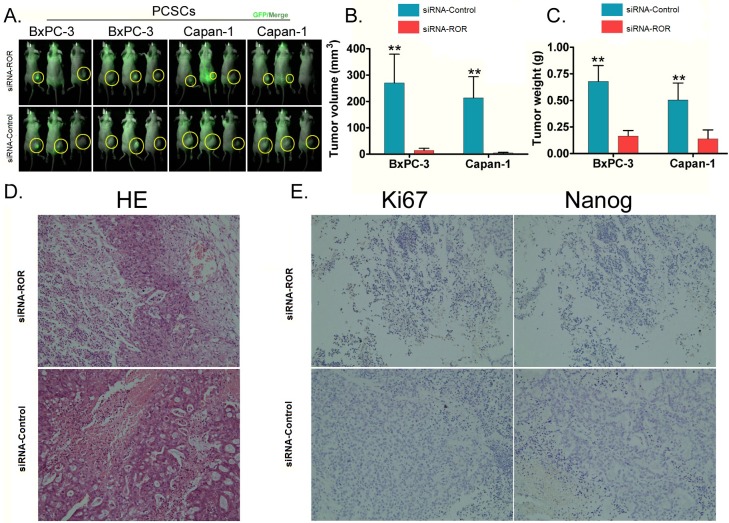
Inhibition of endogenous ROR expression by siRNA can reduce the tumorigenicity of PCSCs in nude mice **A.** to test the effect of the inhibition of endogenous ROR on the tumorigenicity of PCSCs, siRNA-ROR-transfected PCSCs and siRNA-Control-transfected PCSCs were subcutaneously inoculated in nude mice. Five weeks after inoculation, varying sizes of subcutaneous tumors appeared on the back of mice in both groups. For each tumor tissue, a GFP signal was detected using a small animal *in vivo* imaging system, however, there were significant differences in the tumor sizes. **B.** The volume of the subcutaneous tumors generated by PCSCs expressing siRNA-ROR in the nude mice were all significantly smaller than those generated by PCSCs expressing siRNA-Control (** *p* < 0.01; *n* = 3). **C.** The weights of the subcutaneous tumors generated by PCSCs expressing siRNA-ROR in the nude mice were all significantly smaller than those generated by PCSCs expressing siRNA-Control (** *p* < 0.01; *n* = 3). **D.** Hematoxylin and eosin (HE) staining for histopathology showed that all tumors, irrespective of which group of cells had generated it, were of pancreatic cancer origin. Glandular epithelial-like cells were obviously visible in the tumors, and the cancer cells had various morphologies, showing alveolar or cord shapes. While the nuclei were slightly uneven in size, the nucleolus was clear. The cytoplasm was abundant and had particle-like material. **E.** Immunohistochemistry staining for Ki67 and Nanog. There was a strong positive effect on expression of Ki67 and Nanog in siRNA-ROR-transfected cells but only weak positive effect, or even a negative effect, on expression in siRNA-Control-transfected cells.

To examine the effect of the inhibition of endogenous ROR on the tumorigenic ability of PCSCs *in vivo*, both siRNA-ROR-transfected PCSCs and siRNA-Control-transfected PCSCs were inoculated subcutaneously into nude mice. Both groups of nude mice were fed under the same conditions. Two weeks after injection, nude mice injected with siRNA-Control-transfected PCSCs had an obvious swelling at the injection site, whereas the nude mice injected with siRNA-ROR-transfected PCSCs did not demonstrate any swelling. Five weeks after injection, all of the mice were sacrificed. Although the mice in both groups had varying sizes of subcutaneous tumors on their back, and each tumor tissue was positive for GFP using a small animal *in vivo* imaging system (Figure 5A), the results showed that subcutaneous tumors generated by siRNA-ROR-transfected PCSCs were significantly smaller in both size (*p* < 0.01, Figure 5B) and weight (*p* < 0.01, Figure 5C) than those generated by siRNA- Control -transfected PCSCs.

Histopathological examination revealed that the tumors are of pancreatic origin, irrespective of whether they were derived from siRNA-ROR-PCSCs or siRNA- Control-PCSCs. Glandular epithelial-like cells were obviously visible in the tumors (Figure 5D). The cancer cells had various morphologies, showing alveolar or cord shapes. While the nuclei were slightly uneven in size, the nucleolus was clear. The cytoplasm was abundant and contained particle-like material (Figure 5D). However, the immunohistochemistry results showed that the expression of Nanog and Ki67 protein in the tumor tissues generated by siRNA-ROR-transfected cells was strongly reduced, whereas their expression in tumor tissues generated by siRNA- Control -transfected cells was only weakly reduced or even enhanced (Figure 5E). In summary, these *in vivo* experiments show that when endogenous ROR expression is suppressed in PCSCs, the tumourigenicity of these cells in nude mice is reduced and the expression of Nanog and Ki67 is also downregulated.

## DISCUSSION

ROR is a typical lncRNA that plays important regulatory roles in maintaining stem cell pluripotency, and in the pathogenesis and progression of tumors [14–16]. Sabine et al. found that high expression of ROR promotes the reprogramming of somatic cells into induced pluripotent stem (iPS) cells [17]. In addition, some studies have indicated that the regulation of TGF-β expression by extracellular vesicle-mediated ROR transfer regulates the tolerance of CD133+ liver cancer stem cells to chemotherapeutic drugs [15]. Zhang et al. found that ROR inhibits expression of the cell proliferation inhibitory factor p53 by interacting with the RNA binding protein heterogeneous nuclear ribonucleoprotein I, and thereby promotes breast cancer proliferation [18]. Other studies have indicated that ROR regulates invasion and metastasis by inducing the epithelial-to-mesenchymal transition of ovarian cancer cells [19]. In addition, recent studies by Wang et al. and Chen et al. have shown that the levels of ROR in embryonic stem cells are controlled by endogenous miR-145 [14, 16]. The miR-145 may function as a ‘sponge’-like material, absorbing ROR and regulating cell proliferation [16].

In this study, we tested the expression of ROR in pancreatic carcinoma samples and their non-tumorous tissues. We also identified the function of ROR in PCSCs by applying loss-of-function approaches. The results demonstrated that ROR was upregulated in pancreatic cancer tissues in comparison with adjacent normal pancreatic tissues, and that ROR upregulation correlated with tumor size. Moreover, the overall survival time of patients with lower ROR expression levels was significantly longer than that of patients with moderate or strong ROR expression levels. Furthermore, ROR depletion inhibited cell invasion and cell viability, and induced growth arrest both *in vitro* and *in vivo*. Additionally, ROR suppression led to the promotion of PCSCs apoptosis. These findings suggest that ROR plays a direct role in the modulation of multiple oncogenic properties and pancreatic cancer progression, stimulating new research directions and therapeutic options considering ROR as a novel prognostic marker and therapeutic target in pancreatic cancer.

Inspired by the competitive endogenous RNAs’ regulatory network and increasing evidence suggests that lncRNAs may participate in this regulatory circuitry, we hypothesized that ROR may also serve as a ceRNA and so we searched for potential interactions with miRNAs. RoR has been reported to act as an endogenous sponge of miR-145 in hESCs [16]. We assumed that it had similar effects in PCSCs. To investigate the miRNA-related functions of ROR in pancreatic pathogenesis, we chose miR-145 as a model miRNA for further studies, with a particular focus on the target gene Nanog. Our study had confirmed that Nanog is a direct target of miR-145 (Supplementary Figure 3). Considering the interaction of ROR/miR-145, we therefore hypothesize that ROR may also regulate Nanog expression in PCSCs, which signifies the role of ROR in the tumorigenesis-regulating network.

Meanwhile, it was shown that when miR-145 was bound to ROR, it could negatively silence the expression of an embryonic stem cell pluripotent transcription factor Oct4, and therefore, the pluripotency of the embryonic stem cells was maintained [14, 16, 17, 20]. Similarly, in the present study, when miR-145 was bound to ROR, it could inhibit the expression of the transcription factor Nanog, which have previously been shown to play key roles in maintaining stem cell pluripotency and iPS cell reprogramming [17].

In our pilot experiments, we found that the expression of both Nanog and ROR negatively correlates with miR-145 levels in PCSCs and PCCs. Through bioinformatics, we showed that the 3′UTR of Nanog mRNA has a specific miR-145 binding site. By binding to this site, miR-145 induces Dicer enzyme cleavage of Nanog mRNA to silence Nanog expression. The ROR sequence also contains a miR-145 binding site. Therefore, when both ROR and Nanog exist in the same cell (as in PCSCs), they are in a competition for miR-145 binding.

In this study, the biological role of miR-145 appears to be to silence ROR expression; most miR-145 binds ROR and little is available for silencing Nanog expression. However, when expression of ROR is inhibited with specific siRNA, the level of ROR in PCSCs will decrease dramatically. This means there is more miR-145 available to bind to Nanog, and its expression will be downregulated. The above relationship between ROR, miR-145 and Nanog can be defined as mutually competitive and mutually restrictive. Balancing this “ROR-MiR-145-Nanog” relationship is one of the important mechanisms for PCSCs to maintain their high rates of proliferation, invasion and tumourigenicity.

Indeed, our further experiments confirmed that when endogenous ROR expression is inhibited by specific siRNA in PCSCs, both the *in vitro* proliferation and invasion of PCSCs are significantly reduced. This results from a cell cycle blockage (mainly in the G0/G1 phase) in siRNA-transfected PCSCs. Moreover, when ROR expression is inhibited, tumourigenicity in nude mice is also significantly decreased; not only is tumor formation delayed, but tumor weight and size are also reduced.

In summary, we have identified that a long noncoding RNA, ROR, is up-regulated in PDAC tissues and serves as a prognostic factor in pancreatic cancer patients. The value of ROR as a potential prognostic biomarker and/or therapeutic target in PDAC was supported by findings the epigenetic mechanism of the competitive inhibition of ROR or Nanog expression by miR-145 for inhibiting the proliferation, invasion and tumourigenicity of PCSCs. Silencing ROR expression can enhance the negative regulation of Nanog by miR-145 and effectively reduce the malignant tumor characteristics of PCSCs.

## MATERIALS AND METHODS

### Isolation and *in vitro* expansion of CD24+/CD44+/ CD133+ phenotype cells by flow cytometric activated cell sorting system

Human pancreatic adenocarcinoma cell line Bxpc-3 and Capan-1 was obtained from American Type Culture Collection. The two cultured cell lines were maintained in a humidified 5% CO2 atmosphere at 37°C. Both the cell lines were regularly authenticated by examining their morphology and testing for the absence of mycoplasma contamination (MycoAlert, Lonza, Rockland, ME, USA). CD24+/CD44+/CD133+ subpopulation cells were isolated from the pancreatic cancer cell lines BxPC-3 and Capan-1 using 4μl of the primary monoclonal antibodies (rabbit anti-human CD24-PerCP-Cy5, rabbit anti-human CD44-FITC, mouse anti-human CD133-PE, eBioscience) stored at 4°C in PBS for 30 min in a volume of 1.0 ml. After reaction, the cells were washed twice in PBS, and were isolated and enriched by flow cytometric (BD FACS Aria, BD Bioscience, CA, USA) sorted, incubated at 10°C in PBS for 15 min and then washed twice in PBS. Single cells were plated at 1000 cells/ml in DMEM:F12 (HyClone), supplemented with 10ng/ml basic fibroblast growth factor (bFGF), 10ng/ml epidermal growth factor (EGF), 5μg/ml insulin and 0.5% bovine serum albumin (BSA) (all from Sigma-Aldrich). All CD24+/CD44+/CD133+ cells were cultured in above conditions as non-adherent spherical clusters which were called pancreatic cancer stem cells (PCSCs), while the CD24-/CD44-/CD133- cells were cultured in above conditions which were called normal pancreatic cancer cells (PCCs). All Cells had been cultured on the same conditions until passage 3th before making ulterior experiments.

### Tissue collection

Fresh-frozen and paraffin-embedded pancreatic cancer tissues and corresponding adjacent non-tumorous pancreatic samples were obtained from Chinese patients a Shanghai Tenth People's Hospital between 2010 and 2014. All cases were reviewed by pathologist and histologically confirmed as pancreatic cancer based on histopathological evaluation. Clinical pathology information was available for all samples (Supplementary Table 1). No local or systemic treatment was conducted in these patients before the operation. This study was approved by the Ethics Committee of Fudan University Shanghai Cancer Center, Shanghai, China, and written informed consent was obtained from each participant, in accordance with the institutional guidelines of our hospital.

### RNA extraction and quantitative real-time PCR analysis

Total RNA was isolated from each cell type using the TRIzol Reagent (Invitrogen), according to the manufacturer's protocol. RNA samples were treated with DNase I (Sigma-Aldrich), and quantified and reverse-transcribed into cDNA using the ReverTra Ace-α First Strand cDNA SynthesisKit (TOYOBO). Quantitative real-time PCR (qRTPCR) was conducted using a RealPlex4 real-time PCR detection system (Eppendorf, Germany) with SYBR Green Realtime PCR Master MIX (TOYOBO). qRT-PCR amplification was performed over 40 cycles of denaturation at 95°C for 15 s and annealing at 58°C for 45 s, and the target cDNA was measured using the relative quantification method. A comparative threshold cycle (Ct) was used to determine relative gene expression normalized to the expression of 18S rRNA. For each sample, Ct values were normalized using the formula: ΔCt = Ct_genes - Ct_18S RNA. Relative expression levels were calculated using the formula: ΔΔCt = ΔCt_all_groups - ΔCt_blankcontrol_group. Values used to plot relative gene expression were calculated using the expression, 2-ΔΔCt. Primers used for cDNA amplification are shown in Supplementary Table 2.

### Recombinant lentivirus generation and injected cells

The recombinant lentivirus of siRNA-ROR and siRNA-Control were packaged and purchased from GenePharma Co.Ltd, Shanghai, China. Two different siRNA against linc-RoR (5′to 3′); siRNA linc-ROR-1: GGAGAGGAAGCCTGAGAGT, and siRNA linc-ROR-2: GGTTAAAGACACAGGGGAA. The corresponding viruses were named Ltv-siRNA-ROR or Ltv-siRNA-Control. Co-transfection of PCSCs was conducted to use 1×109 pfU/ml Ltv-siRNA-ROR or Ltv-siRNa-Control lentivirus, respectively, according to the manufacturer's protocol. The cells were seeded in a six-well plate in DMEM:F12 (HyClone), supplemented with 10ng/ml bFGF, 10 ng/ml EGF, 5μg/ml insulin and 0.5% BSA (all from Sigma-Aldrich), at 37°C in a humidified atmoshpere of air containing 5%CO2, until 80% confluent.

### Luciferase report assay

The cells were seeded at 3×104/well in 48-well plates and co-transfected with 400ng of pRNAT-CMV32-miR-145 or pRNAT-CMV32-Mut-miR-145 or pRNAT-CMV32, 20ng of pGL3cm-ROR-2037-2059 or pGL3cm-ROR-mut-2037-2059 or pGL3cm-Nanog-3UTR or pGL-Nanog-Mut-3UTR or pRL-TK (Progema, Madison, USA) using Lipofectamine 2000 Reagent according to the manufacturer's protocol. After 48hr transfection, luciferase activity was measured using the dual-luciferase reporter assay system (Progema, Madison, USA).

### Northern blotting analysis

For all groups, 20μg of good quality total RNA was analyzed on a 7.5M ureum 12% PAA denaturing gel and transferred to a Hybond N+ nylon membrane (Amersham, Freiburg, Germany). Membranes were crosslinked using UV light for 30s at 1200 mjoule/cm2. Hybridization was performed with the miR-145 or ROR antisense starfire probe, to detect the miR-145 or ROR fragments according to the instruction of the manufacturer. After washing, membranes were exposed for 20-40hr to Kodak XAR-5 films (Sigma-Aldrich Chemical). As a positive control, all membranes were hybridized with a human U6 snRNA probe, 5′-GCAGGGGCCATGCTAATCTTCTCTGTATCG-3′. Exposure times for the U6 control probe varied between 15 and 30min.

### RNA fluorescence *in situ* hybridization (FISH)

The cells were deparaffinized and air-dried for 10 min. Slides were treated with proteinase K (Roche) at 37°C for 3 min. Generally, four different concentrations of proteinase K were used (5, 10, 15, and 20 μg/ml in TBS) for each cell sample. After being washed with PBS, slides were incubated with 1 ng/ul DIG-labeled probe (anti-sense or sense) in a hybridization solution consisting of 5×Denhardt's solution, 2×SSC, 10% dextran, 30% formamide, 1mg/ml t-RNA, and 2mg/ml fish sperm DNA overnight at 42°C after being washed. Positive cells were visualized with anti-DIG-labeled Cy3 (Roche) for 60 min in 0.1 M maleic acid buffer containing 0.15 M NaCl, 2% blocking buffer, and 1% Triton X-100.

### Western blotting analysis

Total proteins extracts of each group cells were resolved by 12% SDS-PAGE and transferred on PVDF (Millipore) membranes. After blocking, the PVDF membranes were washed 4 times for 15min with TBST at room temperature and incubated with primary antibody. Following extensive washing, membranes were incubated with secondary peroxidase-linked goat anti-rabbit IgG (1:1000, Santa Cruz) for 1h. After washing 4 times for 15min with TBST at room temperature once more, the immunoreactivity was visualized by enhanced chemiluminescence (ECL kit, Pierce Biotechnology), and membranes were exposed to Kodak XAR-5 films (Sigma-Aldrich Chemical).

### Flow cytometric (FCM) analysis of cell cycle by PI staining

Each group cells was seeded at 3×10^5^ per well in 6-well plates and cultrued until 85% confluent. Each group cells was washed by PBS on three times, then were collected by centifugation (Allegra X-22R, Beckman Coulter) at 1000g for 5min. The cell pellets were the resuspended in 1mL of PBS, fixed in 70% ice-cold ethanol, and ketp in a freezer more than 48h. Before flow cytometric analysis, The fixed cells were centrifuged, washed twice with PBS, and resuspended in PI staining solution (Sigma Chemicals) containing 50μL/mL PI and 250μg/mL RNase A (Sigma Chemicals). The cell suspension, which was hidden from light, was incubated for 30min at 4°C and analyzed using the FACS (BD FACSAria, BD Biosciences). A total of 20,000 events were acquired for analysis using CellQuest software.

### Cell proliferation assay

Each group cells was seeded at 2×103 per well in 96-well plates. After cells were transfected 72 h, 20 ul of Cell Counting Kit-8 (Dojindo Molecular Technologies, Inc., Gaithersburg, MD) was added into each well, which counted the number of living cells using WST-8. The plate was allowed to stand for 2 h at 37°Cand the absorbance at 450 nm was recorded.

### Soft agar colony formation assay

Soft Agar Assays were constructed in 6-well plates. The base layer of each well consisted of 2.0mL with final concentrations of 1×media and 0.6% low melting point agarose. Plates were chilled at 4°C until solid. Upon this, a 1.0 ml growth agar layer was poured, consisting of 1×104 cells suspended in 1× media and 0.3% low melting point agarose. Plates were again chilled at 4°C until the growth layer congealed. An additional 1.0 ml of 1× media without agarose was added on top of the growth layer on day 0 and again on day 15 of growth. Cells were allowed to grow at 37°C for 1 month and total colonies counted. Assays were repeated a total of 3 times.

### Transwell migration assay

Cells (2×10^3^) were resuspended in 200μl of serum-free medium and seeded on the top chamber of the 8.0μm pore, 6.5mm polycarbonate transwell filters (Corning). The full medium (600μl) containing 10% FBS was added to the bottom chamber. The cells were allowed to migrate for 24h at 37°C in a humidified incubator with 5% CO2. The cells attached to the lower surface of membrane were fixed in 4% paraformaldehyde at room temperature for 30mins and stained with 4,6-diamidino-2-phenylindole (DAPI) (C1002, Beyotime Inst Biotech, China), and the number of cells on the lower surface of the filters was counted under the microscope. A total of 5 fields were counted for each transwell filter.

### Histopathology

Briefly, the tissues were stained with hematoxylin and eosin (HE) for analysis by histopathology. Briefly, fresh tissues were washed 3 times with PBS, fixed in 4% paraformaldehyde (Sigma-Aldrich, St. Louis, USA) for 30 min, dehydrated through a graded series of ethanol, vitrified in xylene, and embedded in paraffin. Next, 6-μm thick sections were cut in serial succession and stained with HE.

### Immunohistochemical stain assay

Briefly, the tissues were embedded in paraffin, made in 4 μm slices, tissue sections was dewaxed (4 μm), rinsed with 3% phosphate buffer, and were under microwave heat repairing. The first antibody was added and incubated, horseradish peroxidase conjugated second antibody was added and incubated, ABC chromogenic reagent was used for the color reaction. The first antibody anti-nanog (1:1000) and anti-ki67 (1:1000) were purchased from Cell Signaling Technology, Inc (CST). Meanwhile, the PBS (pH 7.4) was used as a negative control instead of the first antibody. Five randomly vision (200×) of each tissue section was observed and analyzed by IPP software.

### Co-Immunoprecipitation (co-IP) of RNA-processor proteins and associated non-coding RNAs

Brief, all group cells were lysed (500μL per plate) in a modified cell lysis buffer for western and IP (20mM Tris, pH7.5, 150mM NaCl, 1% Triton X-100, 1mM EDTA, sodium pyrophosphate, β-glycerophosphate, Na3VO4 and leupeptin) (Beyotime institute of Biotechnology). After lysis, the each sample was centrifuged to clear the lysate of the insoluble debris and preincubated with 20μg protein A agarose beads (Beyotime institute of Biotechnology) by rocking for 30min at 4°C, followed by centrifugation and transfer to a fresh 1.5mL tube. The rabbit anti-human Dicer polyclonal antibody (1:500; santa cruz biotechnology, California, USA) was incubated for 90 min before re-addition of 20μg protein A agarose beads to capture the immune complexes. The agarose beads were washed three times with ice-cold homogenization buffer.

### *In vivo* xenograft experiments

About 1×105 logarithmically growing siRNA-ROR transfected PCSCs or siRNA-Control transfected PCSCs were inoculated BALB/nude/nude mice, respectively. Each experimental group consisted of six mice. After five weeks of observation, the mice were sacrificed and tumors were stripped. The tumor was weighed and its volume was calculated according to the formula: Tumor volume (mm^3^) = (ab2)/2, where a represents the longest axis (mm) and b the shortest axis (mm). Male nude BALB/c nude/nude mice (6-8 weeks old) were obtained from the Animal Research Center of Fudan University, China. This study was approved by the Ethics Committee of Fudan University Shanghai Cancer Center, Shanghai, China. All of the mice experiments were conducted in accordance with the guidelines of the NIH for the care and use of laboratory animals. The study protocol was also approved by the Committee on the Use of Live Animals in Teaching and Research, Fudan University, Shanghai, China.

### Statistical analysis

Each experiment was performed as least three times, and data are shown as the mean ± standard deviation (SD) where applicable, and differences were evaluated using Student's *t* tests. The probability of < 0.05 was considered to be statistically significant.

## SUPPLEMENTARY MATERIAL FIGURES AND TABLES


