# Supplementary material for: ROR functions as a ceRNA to regulate Nanog expression by sponging miR-145 and predicts poor prognosis in pancreatic cancer
Source: Oncotarget. 2015 Dec 2;7(2):1608–18. doi: 10.18632/oncotarget.6450 (PMC4811484; doi:10.18632/oncotarget.6450)
Supplement: Supplementary file 1 [file oncotarget-07-1608-s001.pdf]

ROR functions as a ceRNA to regulate Nanog expression by sponging miR-145 and predicts poor prognosis in pancreatic cancer

Supplementary Material

Supplementary Table 1. The correlation between the lncRNA-ROR expression and clinicopathological factors in resectable PDAC (n=61)

		No. of cases	No. of patients (%)		*P*-Value
			ROR High	ROR Low	
**Age**					
	≤65	34	17(50.0%)	17(50.0%)	1.0000
	＞65	27	14(51.8%)	13(49.2%)	
**Gender**					
	Male	32	15(46.9%)	17(53.1%)	0.0520
	Female	29	14(48.3%)	15(51.7%)	
**Tumor Location**					
	Head	35	18(51.4%)	17(48.6%)	1.0000
	Body and Tail	26	13(50.0%)	13(50.0%)	
**Tumor Size**					
	≤4cm	38	29(76.3%)	9(23.7%)	**0.0297***
	＞4cm	23	16(69.6%)	7(30.4%)	
**Nodal Metastasis**					
	N0	46	23(50.0%)	23(50.0%)	1.0000
	N1-N3	15	8(69.6%)	7(30.4%)	
**CA19-9(IU/ml)**					
	≤500	19	9(47.4%)	10(52.6%)	0.6545
	＞500	42	22(52.4%)	20(47.6%)	
**TNM Stage**					
	I and II	43	23(53.5%)	20(46.5%)	0.8245
	III and IV	18	10(55.6%)	8(44.4%)	
**Tumor Differentiation**					
	Well and Moderate	39	20(51.3%)	19(48.7%)	0.1320
	Poor	22	12(54.5%)	10(45.5%)	

Abbreviations: PDAC, pancreatic duct adenocarcinoma.
*This comparison was performed using Student's t-test.

Supplementary Table 2. The qRT-PCR primers used in the study.

	**primers (5'→3')**
**lncRNA-ROR**	CCAGGACAATGAAACCAC (forward)
	TGGAGCAGGTATGAGATT (reverse)
**miR-145**	GTCCAGTTTTCCCAGGAATCCCT (forward)
	GCTGTCAACGATACGCTACCTA (reverse)
**18S rRNA**	CAGCCACCCGAGATTGAGCA (forward)
	TAGTAGCGACGGGCGGTGTG (reverse)

(A)

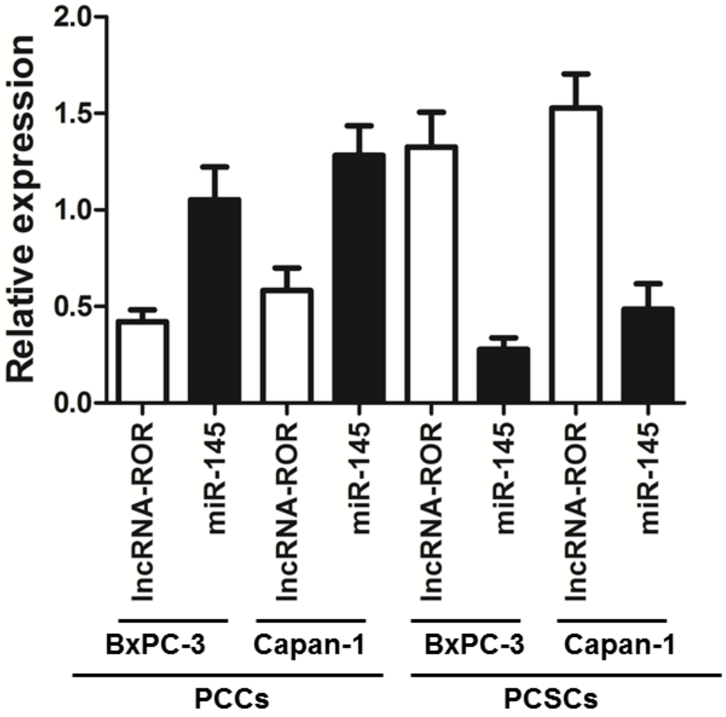

(B)

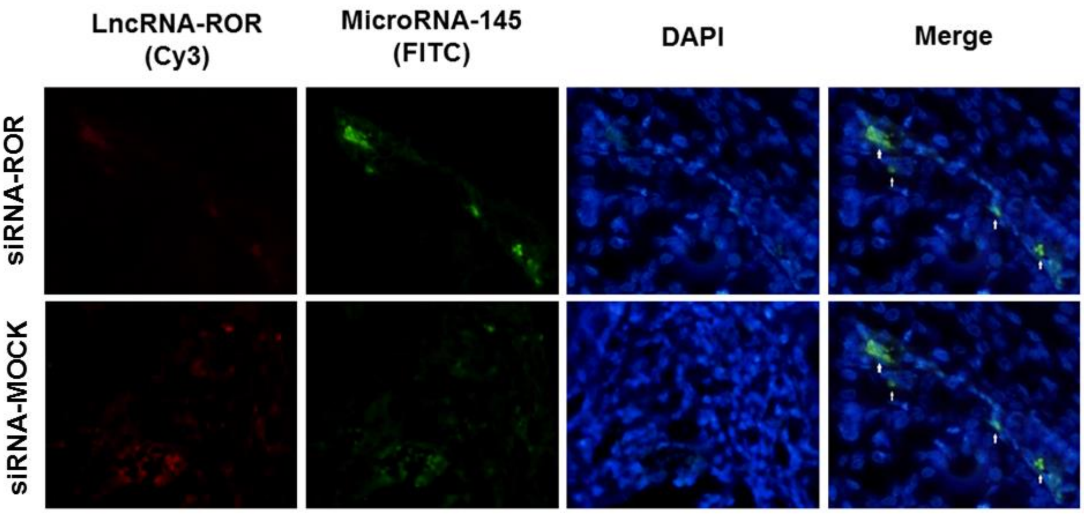

Supplementary Figure 1. **ROR and miR-145 expression are negatively correlated**

We examined the expression of the ROR and miR-145 in PCSCs and PCCs by qRT-PCR **(A)** and analyzed the ROR and miR-145 in serial sections of tissues by in Fluorescence in situ hybridization **(B)**. The result is in agreement with the expression in PCSCs and PCCs, ROR silencing resulted in increased expression of miR-145, so we further confirm that the expression of lncRNA-ROR and microRNA-145 are negatively correlated.

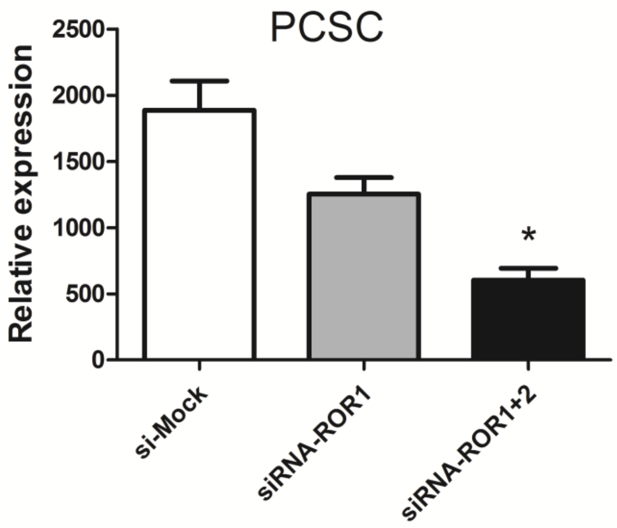

Supplementary Figure 2. The confirmation of ROR expression in transfected pancreatic cancer stem cells.

Although a validated siRNA (siROR-1) of ROR lncRNA has been demonstrated, we chose two siRNAs, siROR-1 and siROR-2, for the construction of the pGIPZROR-shRNA plasmids so as to exclude off-target effects. The pGIPZ ROR-shRNA vectors with an EGFP marker were then packaged into lentiviruses and transduced into human BxPC3 and Capan1 pancreatic cancer stem cells. We used two control cell lines: one with a mock virus carrying the empty vector and one without the virus. Then we detected ROR expression by qRT-PCR. The results indicated that the transfection efficiency of siROR-1 and siROR-2 is higher.

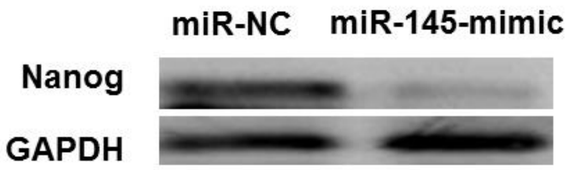

Supplementary Figure 3. Nanog is a direct target of miR-145.

We use western bolt and luciferase reporter assay to confirm the expression between miR-145 and Nanog. To explore the function of miR-145 in the regulation of Nanog, PCSCs were transfected with miR-145 mimic. Then we detect the protein levels of Nanog by western blot, the result showed overexpression of miR-145 significantly inhibited Nanog expression.

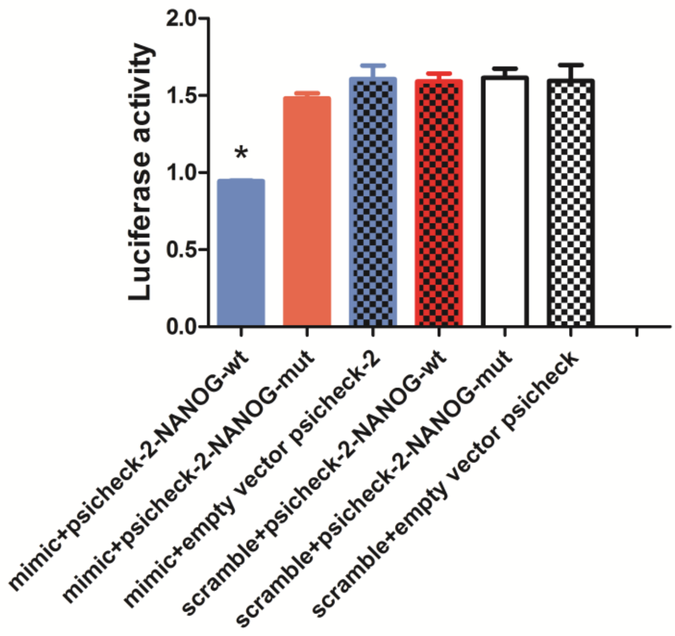

Luciferase reporter assay showed significantly lower luciferase activity compared with the control group when miR-145 and Nanog 785 bp-789 bp were overexpressed in the same cell line. Similarly, the Luciferase activity was significantly lower than the control group ($p < 0.05$). when miR-145 and the Nanog mRNA 3' UTR were simultaneously overexpressed in the same cell line. These results suggested that miR-145 could induce posttranscriptional silencing of its target genes by binding to the Nanog mRNA 3' UTR or specific sites.
